# Factors Influencing the Fungal Diversity on Audio–Visual Materials

**DOI:** 10.3390/microorganisms9122497

**Published:** 2021-12-02

**Authors:** Tereza Branysova, Martina Kracmarova, Michal Durovic, Katerina Demnerova, Hana Stiborova

**Affiliations:** 1 Department of Biochemistry and Microbiology, Faculty of Food and Biochemical Technology, University of Chemistry and Technology, Prague, Technická 3, 16628 Prague 6, Czech Republic; Martina.Kracmarova@vscht.cz (M.K.); Katerina.Demnerova@vscht.cz (K.D.); 2 Department of Chemical Technology of Monument Conservation, Faculty of Chemistry and Technology, University of Chemistry and Technology, Prague, Technická 5, 16628 Prague 6, Czech Republic; Michal.Durovic@vscht.cz

**Keywords:** biodeterioration, audio–visual materials, next-generation sequencing, fungal contamination

## Abstract

The biodeterioration of audio–visual materials is a huge problem, as it can cause incalculable losses. To preserve these cultural heritage objects for future generations, it is necessary to determine the main agents of biodeterioration. This study focuses on identifying fungi, both from the air and smears from photographs and cinematographic films that differ in the type of carrier and binder, using high-throughput sequencing approaches. The alpha diversity measures of communities present on all types of carriers were compared, and a significant difference between cellulose acetate and baryta paper was observed. Next, the locality, type of carrier, and audio–visual material seem to affect the structure of fungal communities. Additionally, a link between the occurrence of the most abundant classes and species on audio–visual materials and air contamination in the archives was proven. In both cases, the most abundant classes were *Agariomycetes*, *Dothideomycetes*, and *Eurotiomycetes*, and approximately half of the 50 most abundant species detected on the audio–visual materials and in the air were identical.

## 1. Introduction

The archiving of historical audio–visual materials, such as film reels or photographs, is essential for the protection of cultural heritage [1]. However, such materials are often contaminated and degraded by microorganisms, with fungi playing a more critical role than bacteria [2]. The reason why these materials are degraded is the presence of a binder that is part of the photographic emulsion—a light-sensitive layer that is responsible for capturing the image. Binders such as gelatine, albumen, or collodion are suitable substrates for the growth of microbial populations [1,3,4]. In addition, the presence of microbes is affected by environmental conditions, such as temperature, humidity, and/or airflow [5]. Therefore, it is necessary to investigate the microbial contamination of materials and the internal air of archives [6].

Studies dealing with the biodeterioration of audio–visual materials stored in archives are limited. Borrego et al. [5] dealt with the influence of air quality on the biodegradation of photographs. However, the identification of microorganisms in this study was only performed by culture-based methods, which are insufficient for a complete capture of microorganisms [7,8]. The very low percentage (approximately 1%) of cultivable species has led to the development of molecular biological methods [9,10]. These methods are used in the field of metagenomics, which is becoming increasingly popular thanks to the development of next-generation sequencing (NGS) [9,11].

In the field of cultural heritage objects, several studies have already successfully used NGS. For example, recent studies include Antonelli et al. [12], who evaluated historical wooden objects, Torralba et al. [13], who focused on sculptures and canvas, Bai et al. [14], who dealt with stone objects, and Migliore et al. [15], who examined the contamination of historical parchments.

However, with audio–visual materials, there is still a lack of sequencing studies. Only Szulc et al. [16] used Illumina MiSeq sequencing to directly analyse microbial diversity in historical gelatine photographs. Nevertheless, this study only focused on one type of photosensitive layer and did not include air analysis. Thus far, no comprehensive study has used NGS to analyse the fungal contamination of audio–visual materials with different photosensitive layers while at the same time including air analysis of the archive in which they are housed.

This work aims to identify fungi in selected state archives in the Czech Republic (State Regional Archives Litoměřice and Prague-Chodovec and State District Archives Nepomuk and Hradištko), not only those found on photographs and film reels but also in archive air. Around 2000 amplicon sequence variants (ASVs) of fungi were identified. Our point of interest was also to evaluate several factors (binder, carrier, type of audio–visual material, and locality) that could influence changes in the structure of fungi communities. We hypothesised that the locality would have the greatest influence, due to different environmental conditions, along with the binder, being the top part of audio–visual materials. We also expected a correlation between species identified in the air and on the audio–visual materials, as the air tends to be cited as a main reason for the contamination of audio–visual materials.

## 2. Materials and Methods

### 2.1. Archives and Audio–Visual Materials Stored Inside

Audio–visual and air samples were taken in four archives: State Regional Archives Litoměřice and Prague-Chodovec and State District Archives Nepomuk and Hradištko. Storage conditions were determined in each archive. The temperature and relative humidity inside archives were measured throughout the sampling process. The average values obtained from more than 60 measurements in each archive, together with the types of ventilation systems filters, are shown in Table 1.

In each archive, smears of audio–visual materials with different binders (gelatine, albumen, and collodion) were collected. In total, 51 smears from photographs, 13 smears from cinematographic films, and 21 air samples were collected. Table 2 shows an overview of all analysed audio–visual materials.

### 2.2. Sampling

Before visiting the archives, PTFE Fluoropore membranes with porosity 0.22 µm and diameter 90 mm (Merck Millipore, Germany) were sterilised by autoclaving. The 0.8% saline solution from water for molecular biology, scalpels, scissors, and tweezers were sterilised by gamma rays. For air sampling, a minimum of 4 samples were collected for each archive. Three thousand litres of air were collected on the PTFE Fluoropore membranes using a MAS-100 Eco aeroscope (Merck Millipore, Germany). Next, at least 10 contaminated films and photographs were selected for sampling in each archive. For all photographs and film strips, a relatively equal proportion was selected for sampling. In addition, the materials were microscopically examined prior to sampling to ensure that the part with microorganisms was sampled. The smears were taken from photographic emulsions using sterile polyurethane sponges, which are gentle on the materials (World Bio-products, Woodinville, WA, USA). The smears and PTFE Fluoropore membranes were stored at −20 °C until further analysis.

### 2.3. Extraction of Total Genomic DNA

In the laboratory, 30 mL of gamma-sterilised saline solution was added to the polyurethane sponges. The samples were homogenised for 3 min using a stomacher. The extracts were filtered through filtration cups with porosity 0.2 µm and PES membrane diameter 50 mm (VWR International, Czech Republic), and the PES membranes were cut out. Next, the DNA of captured microorganisms was isolated using a DNeasy PowerWater Kit (Qiagen, Germany). The PTFE Fluoropore membranes from the aeroscope were cut into quarters. Two quarters of each membrane were processed in a similar way to the PES membranes.

### 2.4. DNA Library Preparing and Sequencing

The DNA library for the ITS region was prepared by using specific primers and two-step PCR. The forward primer 5.8S_Fun 5-AACTTTYRRCAAYGGATCWCT-3 and reverse primer ITS4_Fun 5-AGCCTCCGCTTATTGATATGCTTAART-3 [17] were used for amplification of the ITS-2 region (all Sigma-Aldrich, St. Louis, MO, USA). The master mix content and temperature programs were adopted from a study by Kracmarova et al. [17] with a few modifications. Briefly, the first PCR reaction mix contained nuclease-free water, 1 µM of each of the appropriate primers, 0.02 U/µL of KAPA HiFi HotStart ReadyMix (KAPA Biosystems, Wilmington, MA, USA), and 2 µL of template in a total reaction volume of 15 µL. Each sample was made in 8 replicates. The following thermocycling program was used: 5 min of denaturation at 95 °C, followed by 30 cycles of 20 s at 98 °C, 15 s at 50 °C, and 15 s at 72 °C. Final extension was run at 72 °C for 5 min. Next, the replicates were merged and concentrated using the commercial kit Genomic DNA Clean & Concentrator (ZYMO Research, Irvine, CA, USA).

The index PCR reaction mix contained nuclease-free water, 1 µM of each of the appropriate primers, 0.02 U/µL of KAPA HiFi HotStart ReadyMix (KAPA Biosystems, Wilmington, MA, USA), and 1 µL of template in a total reaction volume of 25 µL. The following thermocycling program was used: 5 min of denaturation at 95 °C, followed by 11 cycles of 20 s at 98 °C, 15 s at 50 °C, and 15 s at 72 °C. Final extension was run at 72 °C for 5 min.

Amplicons were analysed after each PCR and concentration step by 1.5% (*w*/*v*) agarose gel electrophoresis (120 V, 60 min). Finally, the samples were sent to the University of Fairbanks, Alaska, where the samples were sequenced using the Illumina MiSeq high-throughput sequencing platform.

### 2.5. Data Processing and Multivariate Statistical Analysis

The taxonomy was assigned to the sequences using the R [18] package DADA2 [19] with a procedure adopted from DADA2 pipeline version 1.16. First, the primer sequences (both forward and reverse reads) were removed. Sequences were then filtered according to the following parameters: truncLen = c(0,0), maxN = 0, maxEE = (2,2), truncQ = 10, and minLen = 50. Subsequently, chimeric sequences in the data set were identified and removed using the consensus method. To avoid potential errors, sequences that differed by only 1 base were merged and the more abundant sequence was retained. The taxonomy was assigned to the amplicon sequence variants (ASVs) using the Unite database [20]. Details of numbers the ASVs in different types of samples shown in Tables S1–S3. Ra-refaction curves were created for all four archives (Figure S1–S4). All sequences obtained were uploaded to the NCBI database under BioProject accession number PRJNA779927.

The ASV dataset was processed using the VEGAN [21] and PHYLOSEQ [22] packages in the programming language R. Prior to all statistical analyses, compositional normalisation was applied to the data. The Shannon diversity index was used to show the alpha biodiversity for each carrier type. After that, the ASVs’ data were transformed by Hellinger and the influence of different factors (locality, binder, carrier, and type of audio–visual material) was determined with permutation multivariate analysis of variance, so-called PERMANOVA, based on Bray–Curtis distance. To visualise the similarities of fungal community structures among different samples, an ordination technique, canonical correspondence analysis (CCA), was conducted. To exclude the effect of locality, this parameter was used as a covariate in the CCA model. To show the relative abundance of fungal classes in the air of archives and on the audio–visual materials, a stacked bar chart was used. Heat maps were created to demonstrate the 50 most abundant species on audio–visual materials and in the air. In addition, Venn diagrams were used to show the distribution of ASVs in the air and on audio–visual materials.

## 3. Results and Discussion

### 3.1. Fungal Diversity on Audio–Visual Materials

**Figure 1:**
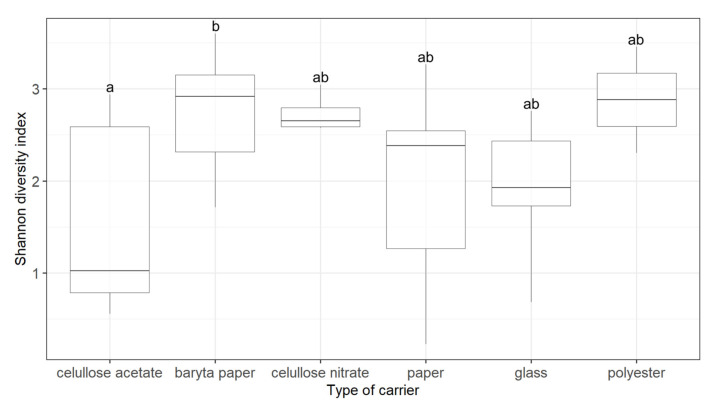
Shannon diversity index showing fungal alpha diversity on audio–visual materials with different types of carriers. Different letters indicate significant differences between treatments (*p*_adj_ ≤ 0.05) and were assigned according to the conducted Tukey HSD test.

The Shannon diversity index showed (Figure 1) that the significant difference (*p*_adj_ < 0.05, Tukey HSD) in fungal biodiversity was only between two types of carriers, namely cellulose acetate and baryta paper. This finding is not surprising, as these two carriers are used for different types of audio–visual material. Cellulose acetate is a typical carrier for film reels [23], while baryta paper has been used for photographic positives [24].

Cellulose acetate films are very susceptible to so-called vinegar syndrome [1], the degradation of acetate to form acetic acid caused by fungal genera such as *Aspergillus*, *Penicillium*, *Fusarium,* or *Trichoderma* [25]. A different carrier, namely polyester (most commonly PET), has been used to address the problems of vinegar syndrome, and polyester is also less biologically susceptible than cellulose acetate [23]. This is because polyester has good strength and flexibility and does not need to be plasticised with additives, which usually act as substrates for microorganisms [26]. Therefore, polyester is perceived as less susceptible to degradation than cellulose acetate, and lower diversity was expected. However, no significant difference (*p*_adj_ = 0.3) in diversity was detected between the cellulose acetate and polyester (Figure 1).

Baryta paper is a carrier that is still used today [16]. The paper itself can serve as a source of energy (carbon or nitrogen) for numerous microorganisms, especially those with cellulolytic activities [27]. Baryta paper is produced by treating paper with barium sulphate mixed with gelatine, starch, or casein, depending on the desired final texture [28]. This mixing with gelatine, starch, or casein does not inhibit the growth of microorganisms; rather, it makes baryta paper well-degradable by microorganisms [29,30,31], as it seems both paper and baryta paper are susceptible to microbial degradation. Our results show that there are not even any significant differences (*p*_adj_ = 0.06) between these two materials in their biodiversity.

### 3.2. Factors Influencing the Structure of Fungal Communities

Locality, carrier, and type of audio–visual material were significantly associated with the structure of the fungal community (*p* < 0.05, PERMANOVA) (Table 3). Most of the variability in fungal community structure was explained by locality (Table 3). The reason why locality played an important role is probably due to the fact that each archive has different storage conditions, such as temperature and humidity (Table 1). These storage conditions are also related to the presence of fungi in the air, which has been identified as one of the main factors affecting the biodeterioration of cultural heritage objects [32]. It is reported that 30 to 50% relative humidity is ideal for preventing fungal growth [33]. This is because only xerophilic fungi are able to grow at lower relative humidity [11]. Conversely, higher relative humidity can promote the growth of more fungal species [33], which can lead to changes in the structure of fungal communities. Furthermore, different fungal species grow at different temperatures, especially in combination with higher relative humidity, and moreover, the appropriate combination of these two factors can lead to a doubling of the number of fungi detected in the air [5]. It is, therefore, important to maintain appropriate storage conditions.

The influence of the binder on the structure of the fungal community was shown, despite it being expected to be insignificant (Table 3). In fact, to date, studies usually only reported what type of binder they examined, whether it was albumen in Puskarova et al. [34] or, more often, gelatine in Bingley et al. [1], Buckova et al. [24], Grbic et al. [35], Sclocchi et al. [4], and Vivar et al. [23]. There is still a lack of studies that consider the binder or type of carrier as factors influencing the structure of fungal communities. In our study, the type of carrier was significantly associated with the fungal community structure (*p* = 0.039).

**Figure 2:**
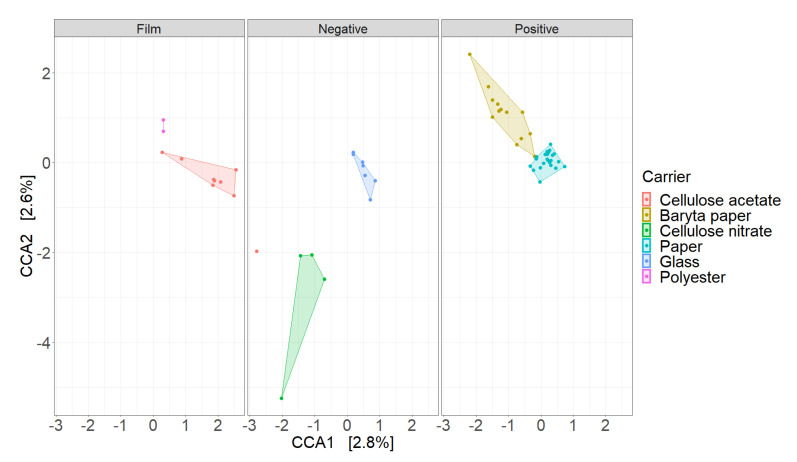
Canonical correspondence analysis (CCA) demonstrates the similarity of fungal community structures between different audio–visual materials (film, negative, and positive) and carriers (cellulose acetate, baryta paper, cellulose nitrate, paper, glass, and polyester).

The CCA ordination (Figure 2) shows the similarity of fungal community structure between different carriers (cellulose acetate, baryta paper, cellulose nitrate, paper, glass, and polyester) of three types of audio–visual materials (film, positive, and negative photography). Polygons representing carriers were separated, indicating that the fungal community structure is influenced by the types of carriers.

For cultural heritage objects other than audio–visual materials, it was verified that the type of material (the type of substrate) can influence the structure of the microbial community. For example, in the case of painted works of art, there may be differences in the structures of microbial communities between paintings on canvas and murals [36]. This is due to the very different composition of the carrier material of paintings on canvas (organic materials—paper, parchment, silk, or wood) and on walls (inorganic materials—stone or brick) [37,38]. Therefore, we believe that the composition of the carrier material could be connected to the structure of microbial populations also in audio–visual materials and its influence should be included, whether in studies of audio–visual materials or other cultural heritage objects.

### 3.3. Relative Abundance of Fungi on Audio–Visual Materials

**Figure 3:**
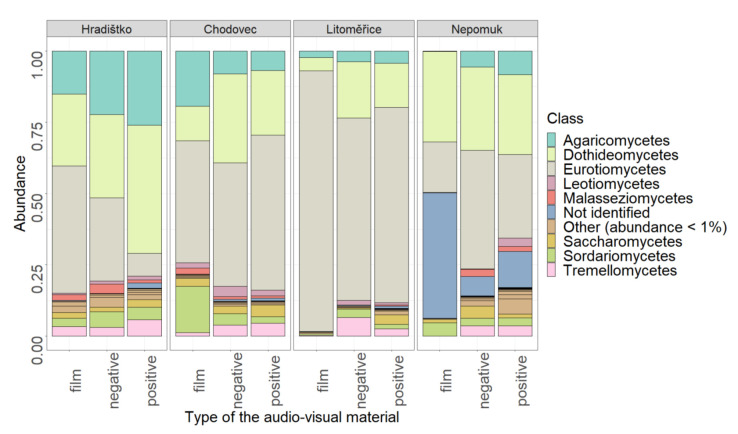
The relative abundance of fungal classes on different types of the audio–visual materials (film, negative, and positive) at different localities (Hradištko, Chodovec, Litoměřice, and Nepomuk).

The relative abundance of fungal taxonomic classes on audio–visual materials is shown in Figure 3. The materials were mainly dominated by two fungal classes from the phylum Ascomycota—*Dothideomycetes* and *Eurotiomycetes*. The *Dothideomycetes* included several genera, for instance, *Alternaria* and *Mycosphaerella*, which were among the most abundant. The class *Eurotiomycetes*, which is the most dominant in most cases, for example, included the abundant genera *Penicillium* and *Aspergillus*. The relative abundance of the class *Agaricomycetes* was also not negligible.

If we compare our results with Szulc et al. [16], the only study so far that has used Illumina MiSeq sequencing, several trends can be observed. Szulc et al. [16] showed *Penicillium*, *Aspergillus*, *Chaetomium*, and *Alternaria* to be the predominant genera on photograph samples. On the same samples, there were high relative abundances of the genera *Talaromyces* and *Fusarium*. In our study, *Penicillium*, *Aspergillus*, and *Alternaria* were also among the most abundant classes. However, the genus *Talaromyces* (class *Eurotiomycetes*) only appeared in two localities in our study, Litoměřice and Hradištko, the genus *Chaetomium* (class *Sordariomycetes*) appeared in Chodovec and Hradištko, and the genus *Fusarium* (class *Sordariomycetes*) appeared only on two audio–visual materials in Nepomuk.

**Figure 4:**
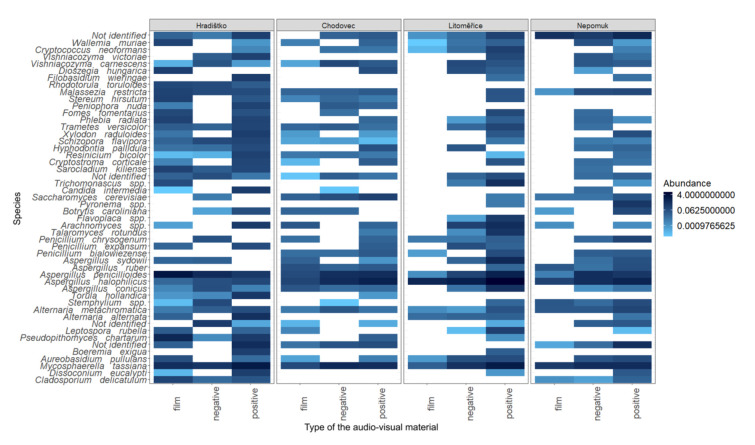
Heat map of 50 most abundant fungal species on the audio–visual materials (film, negative, and positive) at the different localities (Hradištko, Chodovec, Litoměřice, and Nepomuk). A white field indicates the absence of the corresponding species in the sample.

To deepen current knowledge on the contamination of audio–visual materials, the relative abundance of the 50 most prevalent species was displayed on a heatmap (Figure 4). Across all localities and all sampled audio–visual materials, the most abundant species were *Alternaria* *metachromatica*, *A. alternata*, *Aspergillus sydowii*, *A. ruber*, *A. penicillioides*, *A. halophilicus*, *A. conicus*, *Aureobasidium pullulans*, *Cladosporium delicatulum*, *Cryptococcus neoformans*, *Penicillium chrysogenum*, *P. expansum*, and *P. bialowiezense*. All these species belong to fungal genera with proteolytic [25] and cellulolytic [39,40] activities, and therefore are particularly hazardous to audio–visual materials.

Interestingly, some species, for example, *Cladosporium delicatulum* and *Aspergillus ruber*, were not detected at all localities. *Cladosporium delicatulum* was absent at two localities (Chodovec and Litoměřice), and *Aspergillus ruber* was not detected in Hradištko (Figure 4). This might indicate that some genera are not associated with the type of audio–visual material but rather with the air contamination and the storage conditions in specific archives.

### 3.4. Fungal Relative Abundance in the Air of Archives

**Figure 5:**
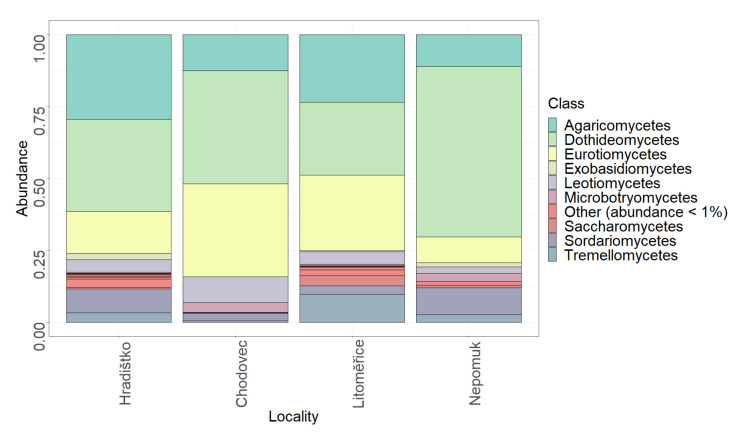
Relative abundance of fungal classes in the air at different localities (Hradištko, Chodovec, Litoměřice, and Nepomuk).

The relative abundance of fungal classes occurring in the air of four archives is displayed in Figure 5. There were nine classes with a relative abundance higher than 1%. The most abundant classes were the same for air as for the audio–visual materials, i.e., *Agaricomycetes*, *Dothideomycetes*, and *Eurotiomycetes*.

Borrego et al. [5] identified several fungal species in the air of two archives. In particular, one of the archives was predominated by the species *Cladosporium* (*Dothideomycetes*), while the other archive was dominated by the genus *Penicillium* (*Eurotiomycetes*). Other identified genera included *Aspergillus* (*Eurotiomycetes*), *Geotrichum* (*Saccharomycetes*), *Curvularia* (*Dothideomycetes*), *Fusarium*, and *Neurosphora* (both *Sordariomycetes*). However, in our study, only *Cladosporium*, *Penicillium*, and *Aspergillus* were the same as in study of Borrego et al. [5].

**Figure 6:**
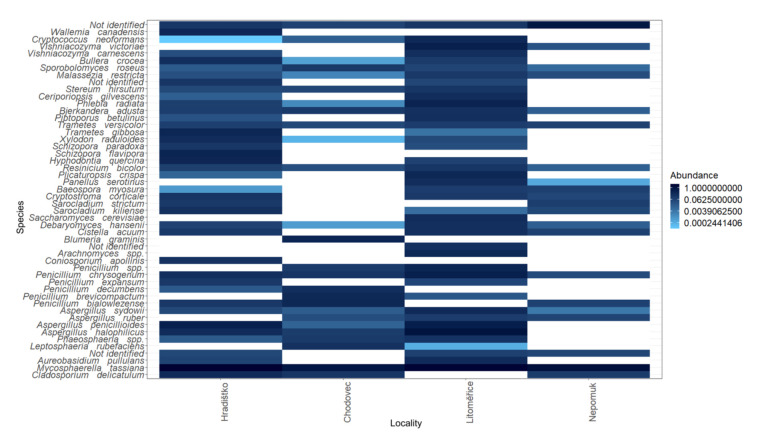
Heat map of 50 most abundant fungal species in the air at different localities (Hradištko, Chodovec, Litoměřice, and Nepomuk). A white field indicates the absence of the corresponding species in the sample.

To address whether there is association between the occurrence of species identified in the air and on the audio–visual materials, a heatmap (Figure 6) of the top 50 fungal species present in the air of archives was created. Approximately half of the 50 species found in the air were also detected on at least one stored audio–visual material (Figure 4). Such findings might reflect a link between air contamination and the contamination of stored audio–visual materials and, therefore, suggests the air in archives as the main source of fungal contamination of audio–visual materials. Since the fungal spores in the air were previously identified as key factors affecting the biodeterioration of various archival materials [5,32], the potential air contamination should be considered prior to the protection of cultural heritage.

Fungi detected on audio–visual materials, though, can reveal information not only about the potential biodeterioration of such materials but also the health risk to archive staff. For instance, *Mycosphaerella tassiana* and *Malassezia restricta* detected on audio–visual materials (Figure 4) were associated with patients having cystic fibrosis or asthma [41] or patients suffering from atopic eczema [42]. 

**Figure 7:**
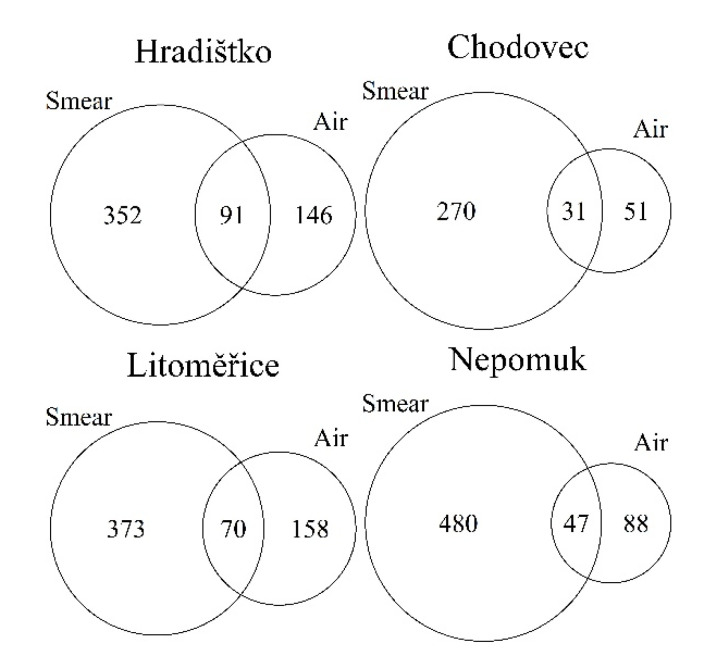
Venn diagrams of distribution of ASVs in air and on audio–visual materials.

Based on the clear link between the fungi identified in air and on audio–visual materials, the total distribution of ASVs in the air and on the audio–visual materials was analysed using Venn diagrams (Figure 7). The ASVs occurring both on smears and in the air confirmed that the air microbiota might be one of the primary causes of audio–visual microbial contamination. At Hradištko, up to 20.5% of the fungi could come from the air, 15.8% at Litoměřice, 10.3% at Chodovec, and 8.9% at Nepomuk. The percentage may be related to the number of ASVs in the air. The lowest number of ASVs (and also the lowest percentage) was at Chodovec. The reason could be the usage of filters in the ventilation systems. The H12 class filters at Chodovec belong to high-efficiency particulate air filters, the so-called HEPA filters [43]. It has been shown that the use of HEPA filters leads to a significant reduction in microorganisms in the air [44]. This class of filters is characterised by its high efficiency (99.99%) for particles > 0.3 μm. In contrast, at Nepomuk were the M5 class of filters. This class belongs to the medium filters group, which has efficiencies between 60 and 90% [45]. However, even there, a lower number of ASVs in the air was found. As already mentioned in Table 1, the archives at Litoměřice and Hradištko do not use filters. Thus, the number of ASV in the air was higher in these archives. 

Only four species were found in the air of all the archives (Hradištko, Chodovec, Litoměřice, and Nepomuk) and on all types of audio–visual materials, namely *Aspergillus sydowii*, *Mycosphaerella tassiana*, *Penicillium chrysogenum,* and *Sporobolomyces roseus*. The genera *Aspergillus* and *Penicillium* are ubiquitous [46], and their species *A*. *sydowii* and *P*. *chrysogenum* have already been identified on film strips in a study by Vivar et al. [23]. In the study by Rojas et al. [47], both *A*. *sydowii* and *P*. *chrysogenum* were identified in the air of a historic building and on the examined materials (books, paintings, and photographs). Moreover, the authors demonstrated cellulose activity and the ability to hydrolyse gelatine in both species, which is unsafe to photographs and film strips.

## 4. Conclusions

To the best of our knowledge, this is the first study assessing several factors influencing the biodiversity and structure of fungal communities on audio–visual materials. Our findings show that fungal community structures differ with the type of carrier and type of audio–visual material. However, locality (determined by storage conditions) explained most of the variability in fungal community structure. As the locality may also be affected to some extent by microorganisms present in the air, we demonstrated overlaps of fungal communities in the air and on audio–visual materials, implying that the primary cause of microbial contamination of audio–visuals is the presence of fungi in the air. It has been shown that 8–20% of fungi present on the materials may originate from contaminated air. This detailed knowledge about fungal communities of audio–visual materials and the association of their structures with the air fungal pollution could help better target the disinfection and protection procedures in museums and archives. Based on our results, it seems that the presence of HEPA filters has a protective effect, as a lower number of ASVs have been identified in the air of the Prague-Chodovec archive. Therefore, further studies could be focused on the comparison of air contamination in the archives with and without HEPA filters.

## Figures and Tables

**Table 1:** Storage conditions in the examined archives.

Archive	Date	Temperature (°C)	Relative Humidity (%)	Air Filters
Litoměřice	11 December 2019	16.7	47.9	none
Hradištko	26 August 2019	24.7	54.6	none
Chodovec	16 July 2020	21.3	50.7	Class H12
Nepomuk	22 September 2020	26.0	45.5	Class M5

**Table 2:** Overview of examined audio–visual materials with different types of photographic emulsion (binders).

Archive	Photographs				Cinematographic Films
	Positive			Negative	Gelatine
	Gelatine	Albumen	Collodion	Gelatine	
Litoměřice	7	5	6	3	1
Hradištko	7	2	1	3	7
Chodovec	2	1	1	3	1
Nepomuk	2	3	1	3	1

**Table 3:** The influence of various factors on the structure of the fungal community (PERMANOVA). Significant (*p* ≤ 0.05) *p*-values are labelled with an asterisk (*).

Factor	R^2^	*p*-Value
Locality	0.1884	0.001 *
Carrier	0.0837	0.039 *
Binder	0.0285	0.362
Type of audio–visual material	0.0214	0.010 *

## Data Availability

Not applicable.

## Supplementary Materials

The following are available online at https://www.mdpi.com/article/10.3390/microorganisms9122497/s1, Table S1. Number of ASVs at different localities. Table S2. Number of ASVs on different types of audio–visual materials. Table S3. Number of ASVs on different types of carriers. Figure S1. Rarefaction curves of samples from State District Archive Nepomuk. Figure S2. Rarefaction curves of samples from State District Archive Hradištko. Figure S3. Rarefaction curves of samples from State Regional Archives Prague-Chodovec. Figure S4. Rarefaction curves of samples from State Regional Archive Litoměřice.

## References

[1] Bingley G., Verran J. (2013). Counts of fungal spores released during inspection of mouldy cinematographic film and determination of the gelatinolytic activity of predominant isolates. Int. Biodeterior. Biodegrad..

[2] Liu Z.J., Zhang Y.H., Zhang F.Y., Hu C.T., Liu G.L., Pan J. (2018). Microbial Community Analyses of the Deteriorated Storeroom Objects in the Tianjin Museum Using Culture-Independent and Culture-Dependent Approaches. Front. Microbiol..

[3] Cappitelli F., Sorlini C. (2005). From papyrus to compact disc: The microbial deterioration of documentary heritage. Crit. Rev. Microbiol..

[4] Sclocchi M.C., Krakova L., Pinzari F., Colaizzi P., Bicchieri M., Sakova N., Pangallo D. (2017). Microbial Life and Death in a Foxing Stain: A Suggested Mechanism of Photographic Prints Defacement. Microb. Ecol..

[5] Borrego S., Guiamet P., de Saravia S.G., Batistini P., Garcia M., Lavin P., Perdomo I. (2010). The quality of air at archives and the biodeterioration of photographs. Int. Biodeterior. Biodegrad..

[6] Borrego S., Lavin P., Perdomo I., de Saravia S.G., Guiamet P. (2012). Determination of Indoor Air Quality in Archives and Biodeterioration of the Documentary Heritage. ISRN Microbiol..

[7] Ciesielski S., Pokoj T., Klimiuk E. (2010). Cultivation-Dependent and -Independent Characterization of Microbial Community Producing Polyhydroxyalkanoates from Raw Glycerol. J. Microbiol. Biotechnol..

[8] Tepla B., Demnerova K., Stiborova H. (2020). History and microbial biodeterioration of audiovisual materials. J. Cult. Herit..

[9] Otlewska A., Adamiak J., Gutarowska B. (2014). Application of molecular techniques for the assessment of microorganism diversity on cultural heritage objects. Acta Biochim. Pol..

[10] Bodor A., Bounedjoum N., Vincze G.E., Kis A.E., Laczi K., Bende G., Szilagyi A., Kovacs T., Perei K., Rakhely G. (2020). Challenges of unculturable bacteria: Environmental perspectives. Rev. Environ. Sci. Biotechnol..

[11] Branysova T., Tepla B., Demnerova K., Stiborova H., Durovic M. (2021). Biodeterioration of Audio-Visual Materials. Chem. Listy.

[12] Antonelli F., Esposito A., Galotta G., Petriaggi B.D., Piazza S., Romagnoli M., Guerrieri F. (2020). Microbiota in Waterlogged Archaeological Wood: Use of Next-Generation Sequencing to Evaluate the Risk of Biodegradation. Appl. Sci..

[13] Torralba M.G., Kuelbs C., Moncera K.J., Roby R., Nelson K.E. (2020). Characterizing Microbial Signatures on Sculptures and Paintings of Similar Provenance. Microb. Ecol..

[14] Bai F.Y., Chen X.P., Huang J.Z., Lu Y.S., Dong H.Y., Wu Y.H., Song S.L., Yu J., Bai S., Chen Z. (2021). Microbial biofilms on a giant monolithic statue of Buddha: The symbiosis of microorganisms and mosses and implications for bioweathering. Int. Biodeterior. Biodegrad..

[15] Migliore L., Perini N., Mercuri F., Orlanducci S., Rubechini A., Thaller M.C. (2019). Three ancient documents solve the jigsaw of the parchment purple spot deterioration and validate the microbial succession model. Sci. Rep..

[16] Szulc J., Ruman T., Karbowska-Berent J., Kozielec T., Gutarowska B. (2020). Analyses of microorganisms and metabolites diversity on historic photographs using innovative methods. J. Cult. Herit..

[17] Kracmarova M., Karpiskova J., Uhlik O., Strejcek M., Szakova J., Balik J., Demnerova K., Stiborova H. (2020). Microbial Communities in Soils and Endosphere of Solanum tuberosum L. and their Response to Long-Term Fertilization. Microorganisms.

[18] R Core Team R. (2017). A Language and Environment for Statistical Computing in R Foundation for Statistical Computing.

[19] Callahan B.J., McMurdie P.J., Rosen M.J., Han A.W., Johnson A.J.A., Holmes S.P. (2016). DADA2: High-resolution sample inference from Illumina amplicon data. Nat. Methods.

[20] UNITE Community (2019). UNITE General FASTA Release for Fungi 2. Version 18.11.2018. UNITE Community. https://unite.ut.ee/repository.php.

[21] Oksanen J., Blanchet F.G., Kindlt R., Legendre P., O’Hara R.B., Simpson G.L., Solymos P., Stevens M.H.H., Wagner H. (2019). Vegan: Community Ecology Package. R-Package Version 2.5-7. https://cran.r-project.org/web/packages/vegan/index.html.

[22] McMurdie P.J., Holmes S. (2013). phyloseq: An R Package for Reproducible Interactive Analysis and Graphics of Microbiome Census Data. PLoS ONE.

[23] Vivar I., Borrego S., Ellis G., Moreno D.A., Garcia A.M. (2013). Fungal biodeterioration of color cinematographic films of the cultural heritage of Cuba. Int. Biodeterior. Biodegrad..

[24] Buckova M., Puskarova A., Sclocchi M.C., Bicchieri M., Colaizzi P., Pinzari F., Pangallo D. (2014). Co-occurrence of bacteria and fungi and spatial partitioning during photographic materials biodeterioration. Polym. Degrad. Stab..

[25] Mazzoli R., Giuffrida M.G., Pessione E. (2018). Back to the past: “Find the guilty bug-microorganisms involved in the biodeterioration of archeological and historical artifacts”. Appl. Microbiol. Biotechnol..

[26] Lourenco M.J.L., Sampaio J.P. (2009). Microbial deterioration of gelatin emulsion photographs: Differences of susceptibility between black and white and colour materials. Int. Biodeterior. Biodegrad..

[27] Kwasna H., Karbowska-Berent J., Behnke-Borowczyk J. (2020). Effect of Fungi on the Destruction of Historical Parchment and Paper Documents. Pol. J. Environ. Stud..

[28] Cattaneo B., Chelazzi D., Giorgi R., Serena T., Merlo C., Baglioni P. (2008). Physico-chemical characterization and conservation issues of photographs dated between 1890 and 1910. J. Cult. Herit..

[29] Martucci J.F., Ruseckaite R.A. (2009). Biodegradation of three-layer laminate films based on gelatin under indoor soil conditions. Polym. Degrad. Stab..

[30] Khoramnejadian S. (2013). Microbial Degradation of Starch Based Polypropylene. J. Pure Appl. Microbiol..

[31] Savkovic Z., Stupar M., Unkovic N., Ivanovic Z., Blagojevic J., Vukojevic J., Grbic M.L. (2019). In vitro biodegradation potential of airborne *Aspergilli* and *Penicillia*. Sci. Nat..

[32] Okpalanozie O.E., Adebusoye S.A., Troiano F., Catto C., Ilori M.O., Cappitelli F. (2018). Assessment of indoor air environment of a Nigerian museum library and its biodeteriorated books using culture-dependent and -independent techniques. Int. Biodeterior. Biodegrad..

[33] Dannemiller K.C., Weschler C.J., Peccia J. (2017). Fungal and bacterial growth in floor dust at elevated relative humidity levels. Indoor Air.

[34] Puskarova A., Buckova M., Habalova B., Krakova L., Makova A., Pangallo D. (2016). Microbial communities affecting albumen photography heritage: A methodological survey. Sci. Rep..

[35] Grbic M.L., Stupar M., Vukojevic J., Maricic I., Bungur N. (2013). Molds in Museum Environments: Biodeterioration of Art Photographs and Wooden Sculptures. Arch. Biol. Sci..

[36] Soffritti I., D’Accolti M., Lanzoni L., Volta A., Bisi M., Mazzacane S., Caselli E. (2019). The Potential Use of Microorganisms as Restorative Agents: An Update. Sustainability.

[37] Ciferri O. (1999). Microbial degradation of paintings. Appl. Environ. Microbiol..

[38] Pyzik A., Ciuchcinski K., Dziurzynski M., Dziewit L. (2021). The Bad and the Good-Microorganisms in Cultural Heritage Environments-An Update on Biodeterioration and Biotreatment Approaches. Materials.

[39] Borrego S., Molina A., Santana A. (2017). Fungi in Archive Repositories Environments and the Deterioration of the Graphics Documents. EC Microbiol..

[40] Pietrzak K., Puchalski M., Otlewska A., Wrzosek H., Guiamet P., Piotrowska M., Gutarowska B. (2017). Microbial diversity of pre-Columbian archaeological textiles and the effect of silver nanoparticles misting disinfection. J. Cult. Herit..

[41] Rick E.M., Woolnough K.F., Seear P.J., Fairs A., Satchwell J., Richardson M., Monteiro W.R., Craner M., Bourne M., Wardlaw A.J. (2020). The airway fungal microbiome in asthma. Clin. Exp. Allergy.

[42] Sugita T., Suto H., Unno T., Tsuboi R., Ogawa H., Shinoda T., Nishikawa A. (2001). Molecular Analysis of Malassezia Microflora on the Skin of Atopic Dermatitis Patients and Healthy Subjects. J. Clin. Microbiol..

[43] Chen C., Ji W., Zhao B. (2019). Size-dependent efficiencies of ultrafine particle removal of various filter media. Build. Environ..

[44] Eckmanns T., Rüden H., Gastmeier P. (2006). The Influence of High-Efficiency Particulate Air Filtration on Mortality and Fungal Infection among Highly Immunosuppressed Patients: A Systematic Review. J. Infect. Dis..

[45] Liu G., Xiao M., Zhang X., Gal C., Chen X., Liu L., Pan S., Wu J., Tang L., Clements-Croome D. (2017). A review of air filtration technologies for sustainable and healthy building ventilation. Sustain. Cities Soc..

[46] Sobral M.M.C., Faria M.A., Cunha S.C., Ferreira I.M.P.L.V.O. (2018). Toxicological interactions between mycotoxins from ubiquitous fungi: Impact on hepatic and intestinal human epithelial cells. Chemosphere.

[47] Rojas T.I., Aira M.J., Batista A., Cruz I.L., Gonzalez S. (2012). Fungal biodeterioration in historic buildings of Havana (Cuba). Grana.

