# Supplementary material for: Factors Influencing the Fungal Diversity on Audio–Visual Materials
Source: Microorganisms. 2021 Dec 2;9(12):2497. doi: 10.3390/microorganisms9122497 (PMC8709410; doi:10.3390/microorganisms9122497)
Supplement: Supplementary file 1 [file microorganisms-09-02497-s001.zip › microorganisms-1485408-supplementary.pdf]

Table S1. Number of ASVs at different localities.

Locality	Number of ASVs
Litoměřice	601
Hradištko	589
Chodovec	352
Nepomuk	615

Table S2. Number of ASVs on different types of audio-visual materials.

Type of audio-visual material	Number of ASVs
Positive	1013
Negative	406
Film	304

Table S3. Number of ASVs on different types of carriers.

Type of carrier	Number of ASVs
Cellulose acetate	268
Baryta paper	436
Cellulose nitrate	176
Paper	710
Glass	260
Polyester	96

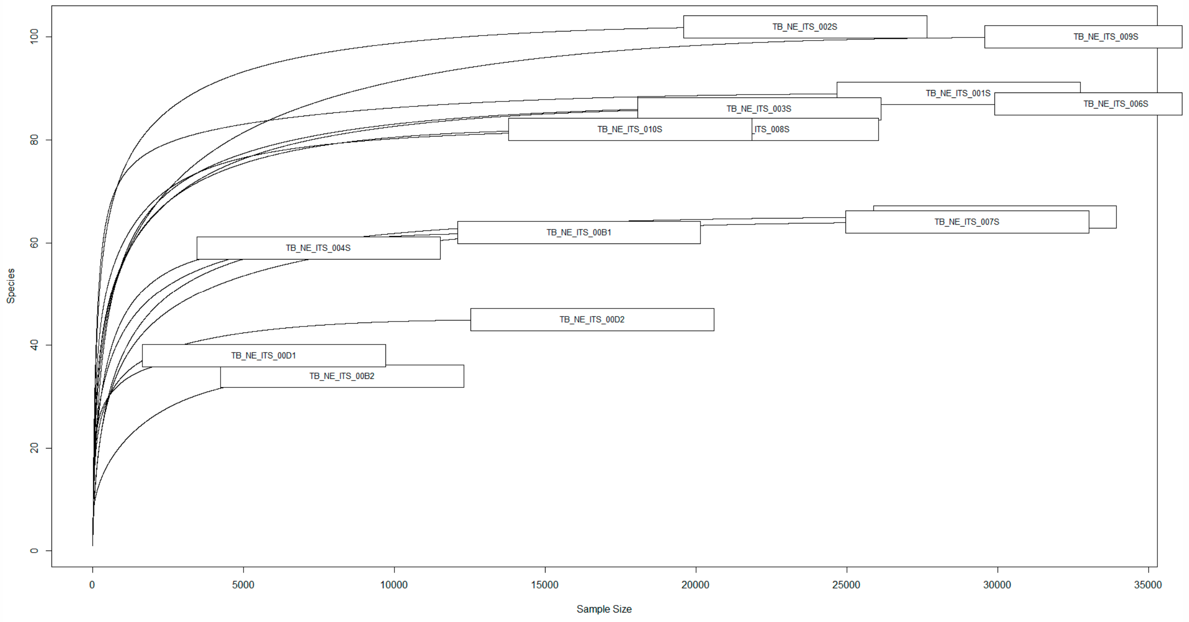

Figure S1. Rarefaction curves of samples from State District Archive Nepomuk.

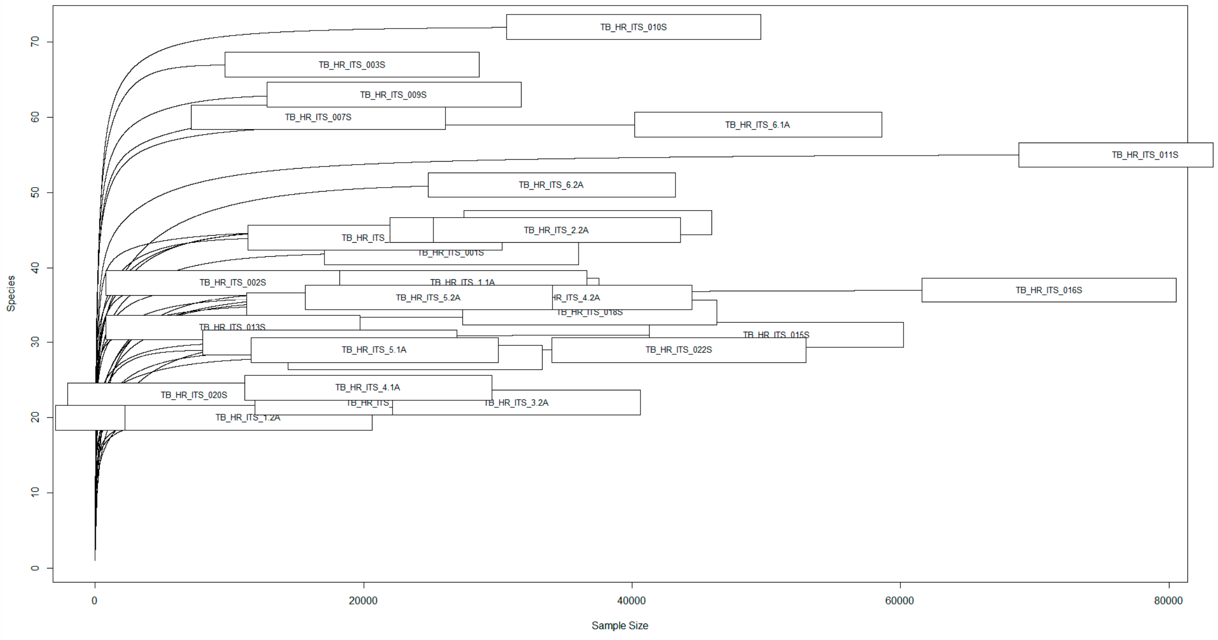

Figure S2. Rarefaction curves of samples from State District Archive Hradištko.

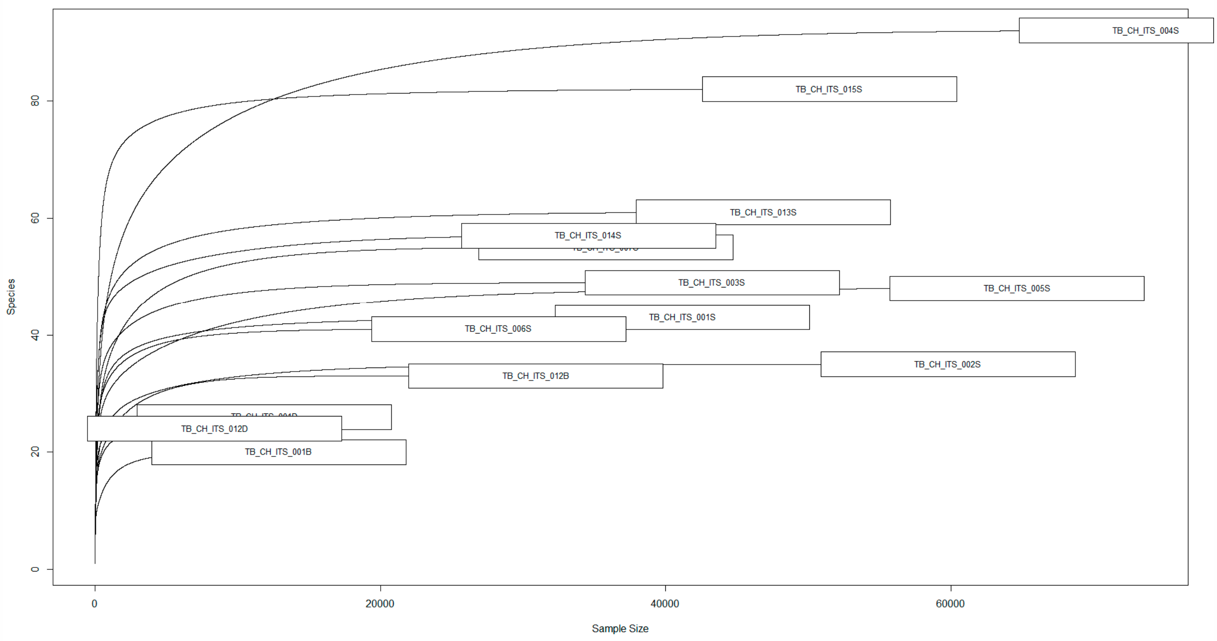

Figure S3. Rarefaction curves of samples from State Regional Archive Prague-Chodovec.

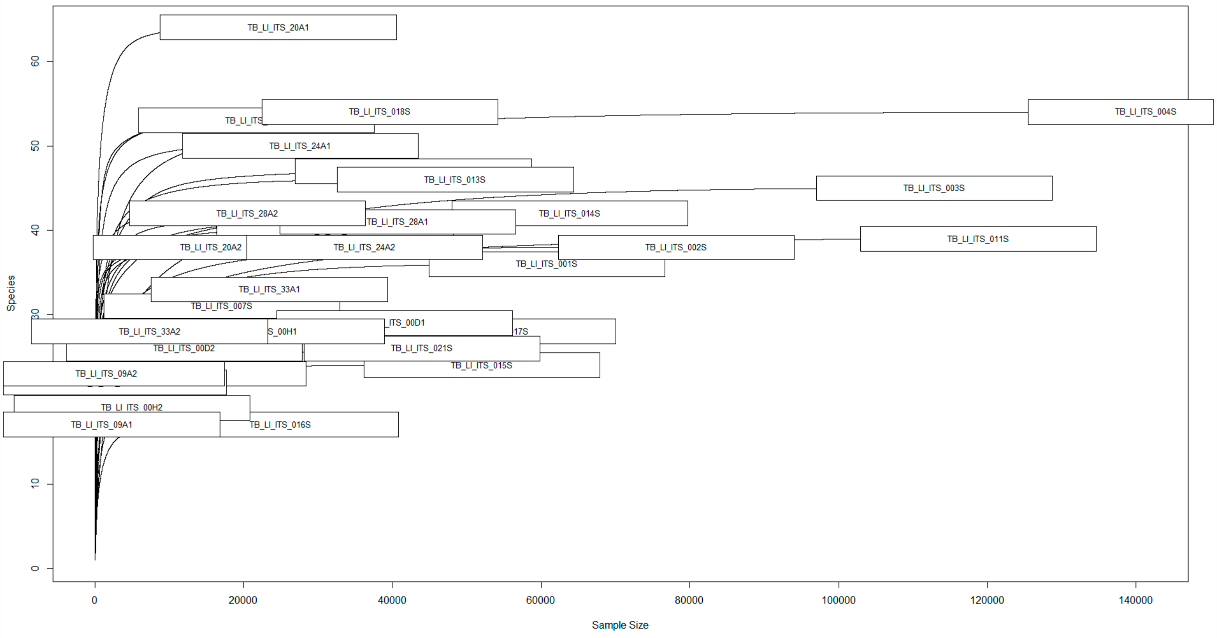

Figure S4. Rarefaction curves of samples from State Regional Archive Litoměřice.
